# Natural Scaffolds for Regenerative Medicine: Direct Determination of Detergents Entrapped in Decellularized Heart Valves

**DOI:** 10.1155/2017/9274135

**Published:** 2017-06-06

**Authors:** Monica Dettin, Annj Zamuner, Filippo Naso, Antonella Monteleone, Michele Spina, Gino Gerosa

**Affiliations:** ^1^Department of Industrial Engineering, University of Padova, Padova, Italy; ^2^Department of Cardiac, Thoracic and Vascular Science, University of Padova, Padova, Italy; ^3^Department of Biomedical Sciences, University of Padova, Padova, Italy

## Abstract

The increasing urgency for replacement of pathological heart valves is a major stimulus for research on alternatives to glutaraldehyde-treated grafts. New xenogeneic acellular heart valve substitutes that can be repopulated by host cells are currently under investigation. Anionic surfactants, including bile acids, have been widely used to eliminate the resident cell components chiefly responsible for the immunogenicity of the tissue, even if detergent toxicity might present limitations to the survival and/or functional expression of the repopulating cells. To date, the determination of residual detergent has been carried out almost exclusively on the washings following cell removal procedures. Here, a novel HPLC-based procedure is proposed for the direct quantification of detergent (cholate, deoxycholate, and taurodeoxycholate) residues entrapped in the scaffold of decellularized porcine aortic and pulmonary valves. The method was demonstrated to be sensitive, reproducible, and extendable to different types of detergent. This assessment also revealed that cell-depleted heart valve scaffolds prepared according to procedures currently considered for clinical use might contain significant amount of surfactant.

## 1. Introduction

At present, the main treatment for valvular heart disease involves surgical replacement with mechanical or biological valve prostheses. Biological prostheses include both cryopreserved homografts (human) and glutaraldehyde fixed xenograft (GFX) of several animal origins (porcine/bovine/equine). The advantages of biological substitutes are the low risks of thrombotic events together with excellent hemodynamic performance, as well as avoiding the need for anticoagulant treatment [1]. Nevertheless, the limited availability of homograft valves; the reduced duration of xenogeneic GFX bioprostheses [2], especially in young patients [3]; and the incomplete biocompatibility of both [4] mean that none could be considered a definitive heart valve substitute. In the search for a safe and unlimited supply of valve substitutes, various proposals have considered the use of xenogeneic scaffolds after resident cell removal. This interest arises from the preservation of their functional architecture, as well as the potential to maintain tissue factors enhancing cellular adhesion while encouraging migration.

In particular, the degeneration process of current bioprostheses has been related to cellular remnants responsible for both the onset of calcification and immune system activation [5] with the likely contribution of other noncellular antigens of extracellular matrix (ECM) origin [6], while the effective removal of such immunogens could obviate the need for glutaraldehyde treatment. Overcoming the cytotoxicity of GFX tissues [6] would in turn open the way to possible repopulation by host cells, enabling xenogeneic viable substitutes to reshape and eventually grow with the host organism [7].

The first attempt at implanting glutaraldehyde-free xenogeneic heart valve scaffolds in humans was carried out with porcine grafts decellularized using a detergent-free procedure: this led to dramatic results with the onset of a hyperacute immune response, intense inflammatory reaction, structural failure, and graft degradation [8, 9]. This was all subsequently attributed to persistence of the major xenogeneic epitope (alpha-Gal), having been only partially removed by the decellularization treatment [10–12]. Conversely, the use of a similar detergent-free procedure applied to cryopreserved human pulmonary valve homografts gave rise to a bioprosthesis that is now adopted in many cardiosurgical centers in ongoing trials of noninferiority with respect to the standard cryopreserved homografts [1]. In a further attempt to overcome the residual immunological response, other research groups started clinical trialing, still ongoing, with pulmonary and aortic homografts decellularized with detergent-based procedures following preclinical animal model investigations [1, 4, 13]. At present, the longest experimental follow-up in an animal model was achieved with detergent-based preparations implanted in pig and sheep [14, 15]. Reported data confirmed the absence of valvular dysfunction or degeneration outcomes, while revealing a well-documented, though partly irregular, repopulation by the host cells.

On the clinical side, Matrix-P® and Matrix-P Plus® are the only detergent-treated xenogeneic valve substitutes tested to date, and conflicting results have been found after implantation in humans. In addition to uneventful follow-up, poor effective viability and activity of engrafting cell elements and limited freedom from graft dysfunction have been reported [1, 16, 17].

In detergent-based scaffold preparation, anionic surfactants including bile acids (cholate, deoxycholate, and taurodeoxycholate) are widely used to remove resident cell and immunogenic components. However, the extent of eventual residual detergent has not been yet fully evaluated despite the possible effects on the long-term survival and/or functional expression of the repopulating cells. In fact, even if cell-scaffold short-term interactions did not reveal evident incompatibility [14, 15], to date the concentration of residual detergents has been determined almost exclusively in the washings following cell removal and not directly in the residual scaffold matrix [18, 19]. Consequently, whether the adverse effects observed on mechanical properties [20], cell adhesion and survival, immune response, and chemokine production [21] might be related to matrix alteration and/or to residual detergent [22] remains undetermined. In particular, interactions with anionic detergents have been reported to decrease the tensile strength of elastin fibers (the major component of blood vessels) and to increase the susceptibility of insoluble elastin and collagen to enzymatic degradation [23–26]. Elastin degradation products in turn are known to promote myofibroblastic and osteogenic differentiation in fibroblasts and to recruit inflammatory cells in vivo [27, 28].

Reliable information on residual detergent could be used to investigate the relationship, if any, between the actual detergent-based preparation procedures and the observed and/or potential drawbacks mentioned above. Additionally, it could be used for end-product quality control in the manufacturing process of the existing acellular bioprosthetic devices currently on the market and considered for clinical use.

To date, very few assay procedures have been proposed for determining residual surfactant concentration within cell-depleted tissues. The only specific colorimetric procedure available is that reported for sodium dodecylsulfate (SDS) determination [29], while other residual surfactants have been evaluated by assays based on dimethylmethylene blue (DMMB), a reagent currently used for generic anionic detergent detection [22, 30, 31] as well as GAG determination. This present report proposes a novel method for the specific quantification of any residual bile acids (cholate, deoxycholate, and taurodeoxycholate) entrapped in the ECM of porcine valve scaffolds as in case of procedures [17, 19, 32, 33] already considered for clinical use or reported as significantly promising for the production of biocompatible cardiovascular substitutes [34, 35]. Both aortic and pulmonary valves are analyzed, recognizing that differences in tissue structure could lead to dissimilar detergent capture [32]. Descriptions are given of our proposed method, which is based on reverse-phase chromatography and UV detection, along with its application to the direct evaluation of residual bile acids released and further separated after proteolytic degradation of the trapping matrix.

## 2. Experimental Part

### 2.1. Materials

Solvents such as trifluoroacetic acid (TFA) and acetonitrile were purchased from Biosolve (Leenderweg, Valkenswaard, Netherlands).

### 2.2. Defatting and Decellularization

The investigation was carried out on leaflets of aortic roots (ARs) and pulmonary trunks (PTs) freshly dissected from the heart of 10- to 12-month-old pigs (Dutch Landrace, Large White) [32]. Three leaflets were excised each from three different whole ARs and PTs, respectively, and subjected to either defatting or four decellularization protocols (a, b, c, and d) as further described. Representative portions (full half) of the three defatted (*n* = 3) and of the three decellularized leaflets (*n* = 12) excised from different AR and PT, respectively, underwent digestion with papain, in order to obtain the final sample ready for analytical chromatography.

Defatting was carried out by suspension of excised cusps under gentle agitation for subsequent 1 h periods at 4°C in 40%, 80%, and 100% (w/v) ethanol followed by 2/1 and 3/1 v/v chloroform/methanol, respectively. Washing and full rehydration were carried out by inverse sequential resuspension in 100%, 80%, and 40% (w/v) ethanol followed by water and lyophilization.

Decellularization was carried out according to previously reported procedures: (a) Matrix-P single detergent sodium deoxycholate-based preparation (*S*-DOC) [17, 33]; (b) combined sodium dodecylsulfate/sodium deoxycholate procedure (*C*-SDS/DOC) [19]; two two-detergent-based treatments: (c) TriCOL method comprising Triton X-100 and sodium cholate [32] and (d) TriTDOC method implementing Triton X-100 and taurodeoxycholate (TDOC) [35]:

- *S*-DOC: suspension in 1% (weight/volume, w/v) deoxycholic acid sodium salt (Fluka, Analytical) at 37°C in presence of 0.25 mL/L fungizone, 100 U/mL penicillin, and 100 mg/mL streptomycin and extraction for two successive 12 h periods under electromagnetic stirring were carried out. In contrast with the original procedure, the resulting scaffolds were also washed for eight additional 12 h periods in sterile physiological saline at room temperature [19]. Samples were then placed in 70% ethanol over two successive 1.5 h periods and finally frozen after harvesting in isotonic saline.
- *C*-SDS/DOC: suspension in 0.5% DOC/0.5% SDS (w/v) (sodium deoxycholate/sodium dodecyl sulfate, SigmaUltra, Sigma-Aldrich) at room temperature and extraction for two successive 12 h periods under agitation were carried out. Phosphate-buffered solution (PBS) containing 0.04% sodium azide was used for ten successive final washings of 12 h each, followed by suspension in isotonic saline under continuous shaking at room temperature.
- TriCOL procedure carried out as previously described [36]: in brief, the procedure included suspension under gentle agitation in hypotonic PBS in presence of protease inhibitors in nonoxidizing conditions; then removal of cell remnants by successive two 10 h extractions in 1% Triton X-100 and one treatment with hypertonic and hypotonic PBS, respectively, followed by two 10 h extractions in 10 mM sodium cholate (0.4% w/v). Extensive final washings were carried out subsequently in PBS, 10% (v/v) isopropanol/isotonic saline, saline, and water.
- Tri-TDOC protocol in part implemented the above (c) TriCOL procedure while replacing the sodium cholate treatment with two successive extraction periods of 8 h each with 4 mM TDOC (0.2% w/v) (sodium taurodeoxycholate, Sigma, St. Louis, USA) in PBS under agitation at room temperature, in darkness. Finally, each sample was sequentially washed for two 1 h periods in sterile PBS, isotonic saline/10% isopropanol (v/v), and isotonic saline, respectively.

### 2.3. Digestion with Papain

The defatted (AR *n* = 3, PT *n* = 3) and the decellularized (AR *n* = 3 and PT *n* = 3 for each a, b, c, and d protocol) half leaflet samples (see above) were blotted with filter paper (Whatman No. 3), weighed, minced, suspended in a volume of deionized water, and heated at 100°C for 2 min for preliminary protein and collagen denaturation. The whole was resuspended in final 0.1 M sodium acetate buffer (15 mg wet weight/mL) and 5 mM ethylenediamine tetra-acetic acid, pH 6.0, containing 5 mM cysteine and digested with papain (from papaya latex (EC 3.4.22.2), Sigma-Aldrich) (2.8 mg/mL) at 60°C for 48 h under continuous agitation. The reaction was supplemented with fresh enzyme every 12 h, the last addition being associated with 100 mM cysteine in 5% v/v of actual reaction mixture. The enzyme was finally inactivated by heating for 5 min at 100°C and the volume of digest normalized to 100 mL.

### 2.4. Development of the Chromatographic Method

The HPLC system comprised a Waters 600 E System Controller equipped with a UV/Vis detector (Model 2487) and the Empower program for the chromogram acquisition and elaboration. The column used was a Vydac C18 218TP54 Protein & Peptide (5 *μ*m, 300 Å, 4.6 × 250 mm, Grace, Columbia, MD, USA). The eluents were as follows: A 0.05% TFA in MilliQ water; B 0.05% TFA in acetonitrile, and they were degassed with helium blowing. Quantitative determination of the detergents in the eluate was carried out with the UV detector at 200 nm for sodium deoxycholate (DOC), sodium cholate (COL), and taurodeoxycholate (TDOC).

The proteolytic digests appeared clear, transparent, and apparently free of suspended particles and were anyway filtered through a 0.45 *μ*m filter. Known amounts of the investigated analytes were added to the digests of defatted aortic and pulmonary leaflets in order to build up calibration curves under conditions similar to those expected in the determination of the specific residual detergent considered after cell removal treatments. The column was loaded with 6 mL of the final normalized digest, at a flow rate of 1 mL/min. The injector was a solvent line (the instrument has four solvent lines) suitably reduced in length. After aspiration of the sample, 12 mL of degassed eluent A was aspirated to wash the line and concentrate the whole sample at the head of the column. The column was flushed for 50 min with 100% eluent A at 1 mL/min flow rate to allow the absorbance to return to the initial value.

**Figure 1:**
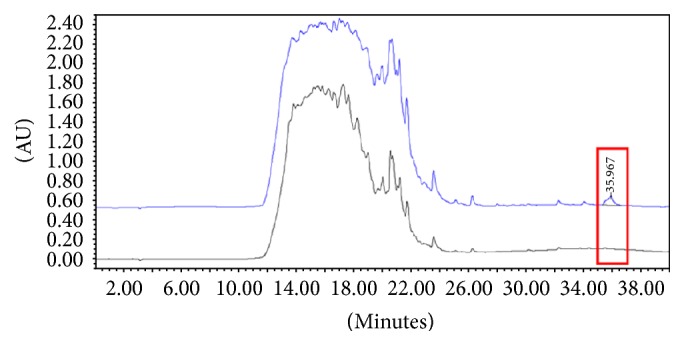
Overlay of chromatograms obtained by running 6 mL of the defatted aortic leaflet digest before (black line) and after (blue line) addition of 0.3 mg deoxycholate (DOC). The two chromatograms were obtained under the following conditions: flow rate, 1 mL/min; eluent A, 0.05% TFA in H_2_O MilliQ; eluent B, 0.05% TFA in CH_3_CN; gradient, from 0% to 80% of eluent B in 40 min; *λ*, 200 nm. AU = optical density.

The isocratic part of the HPLC run enables the elution of the more hydrophilic components of the enzyme-digested sample. The remaining part of the digested sample remains bound to the C18 column: its elution was possible by organic solvent enrichment (acetonitrile) of the eluent phase. The gradient used was developed for each detergent so that the analyte was eluted in a part of the chromatogram particularly free from other components of the digested tissue mixture. For this purpose, aliquots of the defatted leaflets digest were analyzed after enrichment with the detergent under consideration, and the detergent peak was identified after comparison with the chromatographic pattern of the analyte-free digest obtained under the same conditions (Figure 1).

For each detergent, the corresponding peak was registered and its identity confirmed by mass analysis (ESI-ToF Mariner, Applied Biosystems). The mass spectra are given in Supplementary Material (available online at https://doi.org/10.1155/2017/9274135). For the analysis of DOC, a gradient of 0%–80% B over 40 min was used, while the analyses of COL and TDOC were carried out with gradients 0% to 36% of B in 18 min and 36% to 66% of B in 30 min. In both cases, the detector wavelength was set at 200 nm. The runs for all the samples obtained after decellularization treatments were carried out in triplicate by separate injection into the column of three 6 mL aliquots of the previously filtered proteolytic digest.

### 2.5. Preparation of Standards

For the three detergents under consideration, six calibration curves (3 for the pulmonary and 3 for the aortic substrate) were formulated by enriching aliquots (6 mL) of defatted digested samples with increasing amounts of the analyte. The sample concentrations of detergents used to formulate the titration curves are reported in Table 1. The setting of each titration curve was carried out by subsequent injection of one 6 mL aliquot for each individual data point.

### 2.6. Statistical Analysis

Data are expressed as mean ± standard deviation. The titration curves of detergents were calculated through linear regression using Minitab®17 Software. The statistical analysis was performed using 2016 GraphPad Software (GraphPad Software, Inc., La Jolla, CA, USA). Two-tailed Student's* t*-test was used to identify significant differences. A *P* value < 0.05 was considered statistically significant.

## 3. Results

### 3.1. Reproducibility of Retention Times

For each detergent, the analyte peak was eluted at the same reproducible retention time (COL, 31.65 min; DOC, 35.96 min; TDOC, 27.50 min).

### 3.2. Linearity

**Figure 2:**
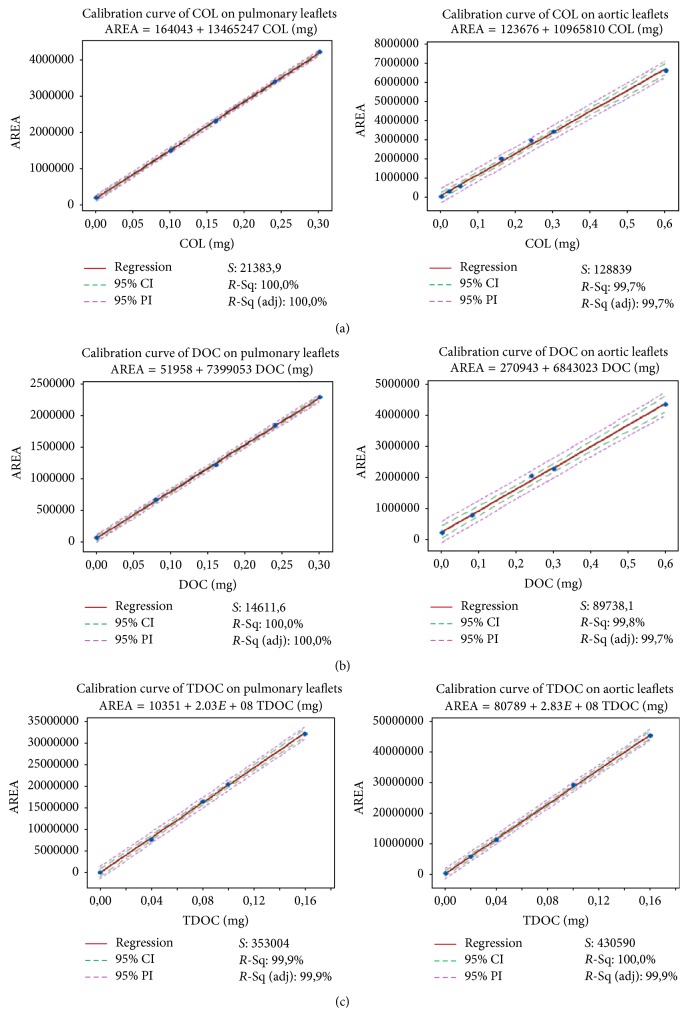
Detergent calibration curves. Each left-right row shows the calibration curves of a single detergent: (a) cholate (COL); (b) deoxycholate (DOC); (c) taurodeoxycholate (TDOC). The curves on the left refer to pulmonary leaflet digest and those on the right to aortic leaflet digest. Area expressed in conventional integration units (CIUs). The coefficient* S* represents the standard deviation of the model's error;* R*-Sq (or *R*^2^) is the percentage of response variable variation explained by its relationship with one or more predictor variables;* R*-Sq (adj) is the percentage of response variable variation explained by its relationship with one or more predictor variables, adjusted for the number of predictors in the model. Confidence intervals (CI) are stated at 95% confidence level. Prediction intervals (PI) of 95% are reported for each regression analysis.

The determination of peak areas of COL, DOC, and TDOC expressed in conventional integration units (CIUs) allowed the formulation of two calibration curves for each detergent, one in the aortic leaflet digest and the other in the pulmonary leaflet digest, shown in Figure 2.

Table 2 presents the resulting figures for the relevant parameters of the calibration curves obtained from regression analysis of the shown data points: slope (*a*), intercept (*b*), correlation coefficient (*r*) and significance level, standard deviation of the slope (*Sa*), and standard deviation of the intercept (*Sb*). In all six instances comprising pulmonary and aortic substrates and three detergents, the high significance level and the *R*^2^ values close to unity are evidence of the reliable proportionality between the peak area and the amount of the analyte added to the defatted sample digest. The value of the 95% confidence limits throughout the range of the regression was also determined and is shown graphically in Figure 2.

### 3.3. Selectivity

Selectivity expresses the ability of a method to assess the analyte in the presence of other components in the system. In this study, the selectivity was examined first by injecting individual solution of each analyte: all detergents under investigation eluted at different retention times. Selectivity was further verified by injecting a combined solution of different detergents. A full separation of these components was achieved and no interference was observed: the copresence of other detergents (Triton X-100, SDS) did not influence either the retention time or the area under the target detergent peak (data not shown).

The method was assessed for the quantification of the DOC content in samples decellularized with both the* S*-DOC and the* C*-SDS/DOC procedures.

### 3.4. Repeatability of Sample Peak Area

**Figure 3:**
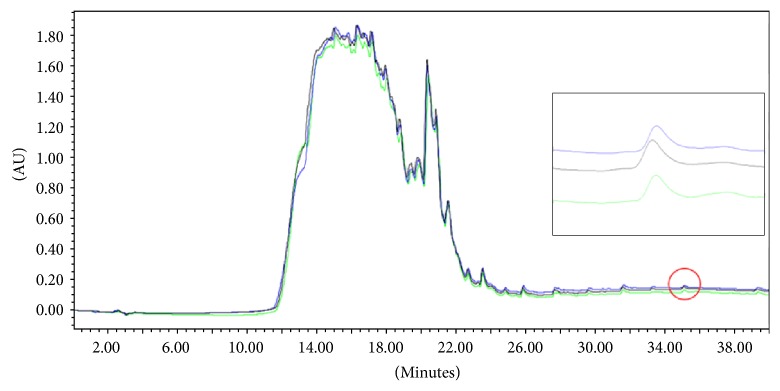
Overlay of the chromatographic traces of three different runs of an identical aliquot of the same SDS-deoxycholate (*S*-DOC) decellularized aortic leaflet digest under the same set of conditions as in Figure 1. AU, optical density; *λ* 200 nm. Right inset: enlargement of the feature circled-peaks corresponding to the detergent.

As one specific example, the chromatograms of three runs carried out by injecting subsequently into the column 6 mL of filtered fresh (not cryopreserved) S-DOC aortic digest for each run are depicted in Figure 3. To express the precision and repeatability of the assay, the coefficient of variation (CV) was calculated, and an average value of 0.04 found. The closeness of CV to zero indicates a low-variance distribution of measures.

### 3.5. Residual Detergent Quantification in Aortic and Pulmonary Leaflet Digests Treated with Different Decellularization Protocols

**Figure 4:**
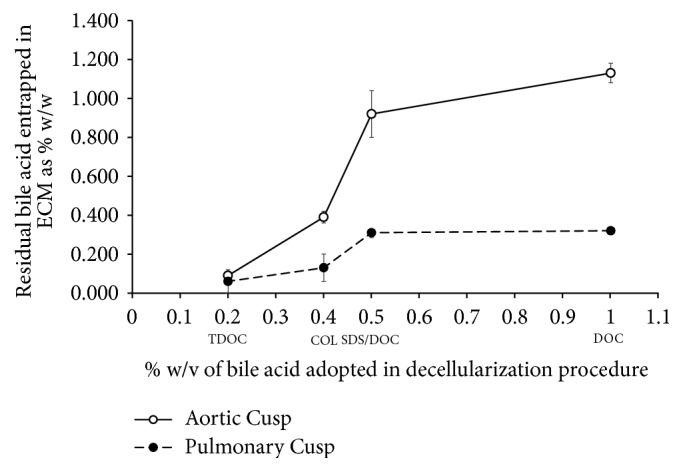
Amount of detergent entrapment in aortic and pulmonary cusps (leaflets) directly related (aortic: *r* = 0.89426, *P* < 0.005; pulmonary: *r* = 0.81582, *P* < 0.02) to the detergent concentration adopted in the decellularization procedure.

The analytical outcomes of the residual detergents detected in the cell-depleted preparations of both aortic and pulmonary leaflets are reported in Table 3. Residual detergent was detected in different concentrations in all the samples. For any particular detergent, its concentration was always found to be significantly lower in the pulmonary preparations relative to the corresponding aortic preparations. This suggests that the difference might be related to the lower thickness of the pulmonary leaflets [32], allowing a better washout of the surfactants. As regards different treatments, the two DOC-based preparations of both aortic and pulmonary leaflets exhibited the highest residual detergent content, less than half of this value being detected in the TriCOL-based preparation and the lowest (about 10 times lower in aortic) in the TriTDOC. The progressively lower residual detergent content found in* C*-DOC versus* S*-DOC (*P* < 0.05), COL versus* C*-DOC (*P* < 0.005), and TDOC versus COL (*P* = 0.0001) was apparently related (aortic: *r* = 0.89426, *P* < 0.005; pulmonary: *r* = 0.81582, *P* < 0.02) to the decreasing detergent concentration adopted in each corresponding procedure (depicted in full in Figure 4), although always used above its critical micelle concentration (CMC). However, the decellularization methods differ from each other in many aspects, such that the different final concentration of entrapped detergents might result from differences in the volumes of cleaning solutions, in the treatment time, in the number and time of washes, and in the type of solvent used.

## 4. Discussion

On the backdrop of ever-wider use of biological valve substitutes and in the interests of patient safety, there is an increasingly urgent need for treatment standardization and end-product characterization. This will enable the monitoring of the possible intake of exogenous compounds and also monitor the persistence of natural components already known to be able to adversely affect the clinical outcome of the implanted devices.

Of several proposed procedures, bile acids and DOC in particular, alone or in combination with other surfactants, have been adopted in the preparation of heart valve substitutes currently under consideration for clinical use [1, 13, 16, 33]. The ability to remove the antigenic determinants of the cell debris holds great promise, since this would eliminate donor-specific immunoresponse in the case of homografts and also avoid the wide use of glutaraldehyde for prevention of rejection-related reactions of xenografts. Although the alpha-Gal xenoreactive epitope has been shown to be completely removed from both porcine aortic and pulmonary valves by the successive action of Triton X-100 and COL [37], a detailed quantitative comparison between the efficacy of different surfactants and/or in combination is not available; furthermore, the amount of detergent remaining within the biological matrix remained uncertain and can only now be determined accurately through the novel method described here.

It is worth noting that bile acids can exist in several states: insoluble (protonated acid), dissolved (monomeric form), and micelles. Switching between these different forms is strongly related to concentration, pH, and temperature [36]. Regarding the decellularization protocols, the main drawback results from the fact that the insoluble form might also be present in a gel-state [38], difficult to remove with simple external washes, and therefore able to be almost irreversibly trapped within the three-dimensional extracellular matrix fiber network.

In fact, for the DOC/SDS-based procedure, a substantial reduction of surfactant leaking from the scaffold can be achieved only after eight changes of washing solution and a total time four times longer than that of the treatment with the detergent itself [19]. Considering these unfavorable release kinetics, complete and/or acceptable detergent depletion is unlikely to be ensured, making it imperative to ascertain the actual value by direct determination within the residual insoluble matrix.

Moreover, with the prospect of full integration of these substitutes after implantation, it remains possible that the natural replacement following further remodeling could release as-yet tightly bound detergent from the implanted matrix, affecting the possible local regeneration activity by damaging the repopulating cells or inducing some adverse reaction by the host.

To the best of the present authors' knowledge, the only report of bile acid surfactant determination within cell-depleted natural scaffolds concerns the evaluation of DOC in decellularized human saphenous vein by the dimethylmethylene blue (DMMB) assay for anionic detergents [30]. However, this assay, also used for SDS determination within decellularized porcine scaffolds [22, 31], is not specific, so interferences with other anionic species present in such a complex biological matrix cannot be excluded. As regards colorimetric assays, here the analyte/s is/are separated by chromatography and individually determined by direct online UV detection in the absence of a revealing reagent, thus avoiding any interaction with possible interfering compounds.

The analytical evidence of sizeable amounts of DOC and other anionic surfactants in ECM preparations (as assessed here) would also provide information about possible positive interferences, as in the case of SDS [39], where DMMB is widely used in assays for GAG evaluation [40, 41]. Indeed, this might explain why the quantitative evaluation of GAG content in DOC decellularized heart valves was found to be twice that determined in the native samples before the cell depletion treatment [42]. On the other hand, the use of DMMB-based assays might present some advantages in nondestructive investigations and in terms of convenience when simple washing fluid mixtures of expected composition are to be analyzed [43].

The method proposed here is certainly capable of separating detergents from all the other anionic species present in such a large protein matrix, allowing correct quantification of each detergent component. Possible risks of lower detergent quantification due to association between detergent and digestion products are avoided through use of a calibration curve derived from detergent peak areas of chromatographic runs in which the analytes are added to the digest of defatted tissue controls.

It should be noted that these decellularization procedures present several differences in the method variables, such as detergent type and concentration, duration, and number of washings. This makes it difficult to relate the amount of bile acid residuals to any specific variable of a method. Even so, the amount of bile acid residues appeared to be related to the surfactant concentration adopted in the particular procedure. Furthermore, for any specific decellularization method, the amount of residual detergent was always higher in aortic samples than in the pulmonary counterparts. This could arise at least in part from the lower thickness of the pulmonary leaflets allowing a better washout, although the effect of some specific tissue features cannot be excluded.

## 5. Conclusions

The analysis of detergents used in decellularization protocols is as yet incomplete since further investigations are needed in order to document possible delayed biological effects of residual detergent. Nevertheless, the work described in present report makes a valuable contribution by describing a direct method for quantifying three detergents used in recently reported decellularization methods. Specifically, it provides a technique for evaluating the concentration/function of residual detergents in biological scaffolds and in addition proposes for the first time an analytical tool for optimizing the several bile-acid-based decellularization procedures proposed. In addition, we maintain that the method can easily be extended to the analysis of other detergents. Furthermore, an efficient decellularization process implementing low bile acid concentrations would also be advisable since this approach would prevent the risk of new potential calcification sites in the treated tissue. In fact, in a physiological environment, unconjugated bile acids (particularly DOC) allow the formation of insoluble calcium salts [44].

## Supplementary Material

Mass spectra of detergents entrapped in decellularized heart valve after tissue digestion and chromatographic separation.

## Figures and Tables

**Table 1:** Standard concentrations of detergents expressed in mg/mL (COL = cholate; DOC = deoxycholate; TDOC = taurodeoxycholate).

*Pulmonary leaflets*	COL mg/mL		*Aortic leaflets*	COL mg/mL	0.0000
		0.0000			0.0033
		0.0166			0.0083
		0.0266			0.0266
		0.0400			0.0400
		0.0500			0.0500
					0.1000

*Pulmonary leaflets*	DOC mg/mL	0.0000	*Aortic leaflets*	DOC mg/mL	0.0000
		0.0133			0.0133
		0.0266			0.0400
		0.0400			0.0500
		0.0500			0.1000

*Pulmonary leaflets*	TDOC mg/mL	0.0000	*Aortic leaflets*	TDOC mg/mL	0.0000
		0.0066			0.0033
		0.0133			0.0066
		0.0166			0.0166
		0.0266			0.0266

**Table 2:** Parameters of calibration curves (see text) for cholate (COL), deoxycholate (DOC), and taurodeoxycholate (TDOC) determination.

*Y* = *ax* + *b*	Pulmonary leaflets			Aortic leaflets		
	COL	DOC	TDOC	COL	DOC	TDOC
*a*	13465247	7399053	2.03*∗*10^8^	10965810	6843023	2.83*∗*10^8^
*b*	164043	51958	10351	123676	270943	80789
*R* ^2^	0.9999 *P* < 0.001	0.9998 *P* < 0.001	0.9994 *P* < 0.001	0.9974 *P* < 0.001	0.9976 *P* < 0.005	0.9996 *P* < 0.001
*Sa*	0.91*∗*10^5^	0.61*∗*10^5^	29.1*∗*10^5^	2.49*∗*10^5^	1.93*∗*10^5^	32.91*∗*10^5^
*Sb*	0.17*∗*10^5^	0.15*∗*10^5^	2.72*∗*10^5^	0.69*∗*10^5^	0.62*∗*10^5^	2.85*∗*10^5^

**Table 3:** Concentration (% w/lyophilized leaflet weight) of residual detergent in decellularized aortic and pulmonary leaflets; single deoxycholate (*S-*DOC), combined deoxycholate/SDS (C-DOC/SDS), triton/cholate (TriCOL), and triton/taurodeoxycholate (Tri-TDOC).

Decellularization procedure	Analyte	Aortic leaflets mg/100 mg	Pulmonary leaflets mg/100 mg	Aortic versus pulmonary
				*P* value
*S*-DOC	DOC	1.13 ± 0.05	0.32 ± 0.005	*P* < 0.0001
*C*-DOC/SDS	DOC	0.92 ± 0.12	0.31 ± 0.02	*P* < 0.001
TriCOL	COL	0.39 ± 0.03	0.13 ± 0.07	*P* < 0.005
Tri-TDOC	TDOC	0.09 ± 0.002	0.06 ± 0.005	*P* < 0.001
