# Corrigendum to “Natural Scaffolds for Regenerative Medicine: Direct Determination of Detergents Entrapped in Decellularized Heart Valves”

**DOI:** 10.1155/2018/2340875

**Published:** 2018-02-20

**Authors:** Monica Dettin, Annj Zamuner, Filippo Naso, Antonella Monteleone, Michele Spina, Gino Gerosa

**Affiliations:** ^1^Department of Industrial Engineering, University of Padova, Padova, Italy; ^2^Department of Cardiac, Thoracic and Vascular Science, University of Padova, Padova, Italy; ^3^Department of Biomedical Sciences, University of Padova, Padova, Italy

In the article titled “Natural Scaffolds for Regenerative Medicine: Direct Determination of Detergents Entrapped in Decellularized Heart Valves” [1], there were errors in the Discussion section which should be corrected as follows:

- “To the best of the present authors' knowledge, the only report of bile acid surfactant determination within cell-depleted natural scaffolds concerns the evaluation of DOC in decellularized human saphenous vein by the dimethylmethylene blue (DMMB) assay for anionic detergents [30]” should be “To the best of the present authors' knowledge, the only report of bile acid surfactant determination within cell-depleted natural scaffolds concerns the evaluation of DOC in decellularized human saphenous vein by the methylene blue (MB) assay for anionic detergents [30].”
- “The analytical evidence of sizeable amounts of DOC and other anionic surfactants in ECM preparations (as assessed here) would also provide information about possible positive interferences, as in the case of SDS [39], where DMMB is widely used in assays for GAG evaluation [40,41]” should be “The analytical evidence of sizeable amounts of DOC and other anionic surfactants in ECM preparations (as assessed here) would also provide information about possible positive interferences of SDS [39] with a reagent similar to MB, the dimethylmethylene blue (DMMB) widely used in assays for GAG evaluation [40,41].”
- “On the other hand, the use of DMMB-based assays might present some advantages in nondestructive investigations and in terms of convenience when simple washing fluid mixtures of expected composition are to be analyzed [43]” should be “On the other hand, the use of MB-based assays might present some advantages in nondestructive investigations and in terms of convenience when simple washing fluid mixtures of expected composition are to be analyzed [43].”
