# Supplementary material for: Natural Scaffolds for Regenerative Medicine: Direct Determination of Detergents Entrapped in Decellularized Heart Valves
Source: Biomed Res Int. 2017 Jun 6;2017:9274135. doi: 10.1155/2017/9274135 (PMC5476881; doi:10.1155/2017/9274135)
Supplement: Supplementary file 1 — Mass spectra of detergents entrapped in decellularized heart valve after tissue digestion and chromatographic separation. [file 9274135.f1.docx]

**Natural scaffolds for regenerative medicine: direct determination of**

**detergents entrapped in decellularized heart valves**

**Monica Dettin, MSc^1*^, Annj Zamuner, MSc^1^, Filippo Naso, MSc^2^, Antonella Monteleone, MSc^2^,**

**Michele Spina, MD, PhD^3^, Gino Gerosa, MD^2^**

1. Department of Industrial Engineering, University of Padova, Padova, Italy.
2. Department of Cardiac, Thoracic and Vascular Science, University of Padova, Padova, Italy.
3. Department of Biomedical Sciences, University of Padova, Padova, Italy.

**Supplementary Information**


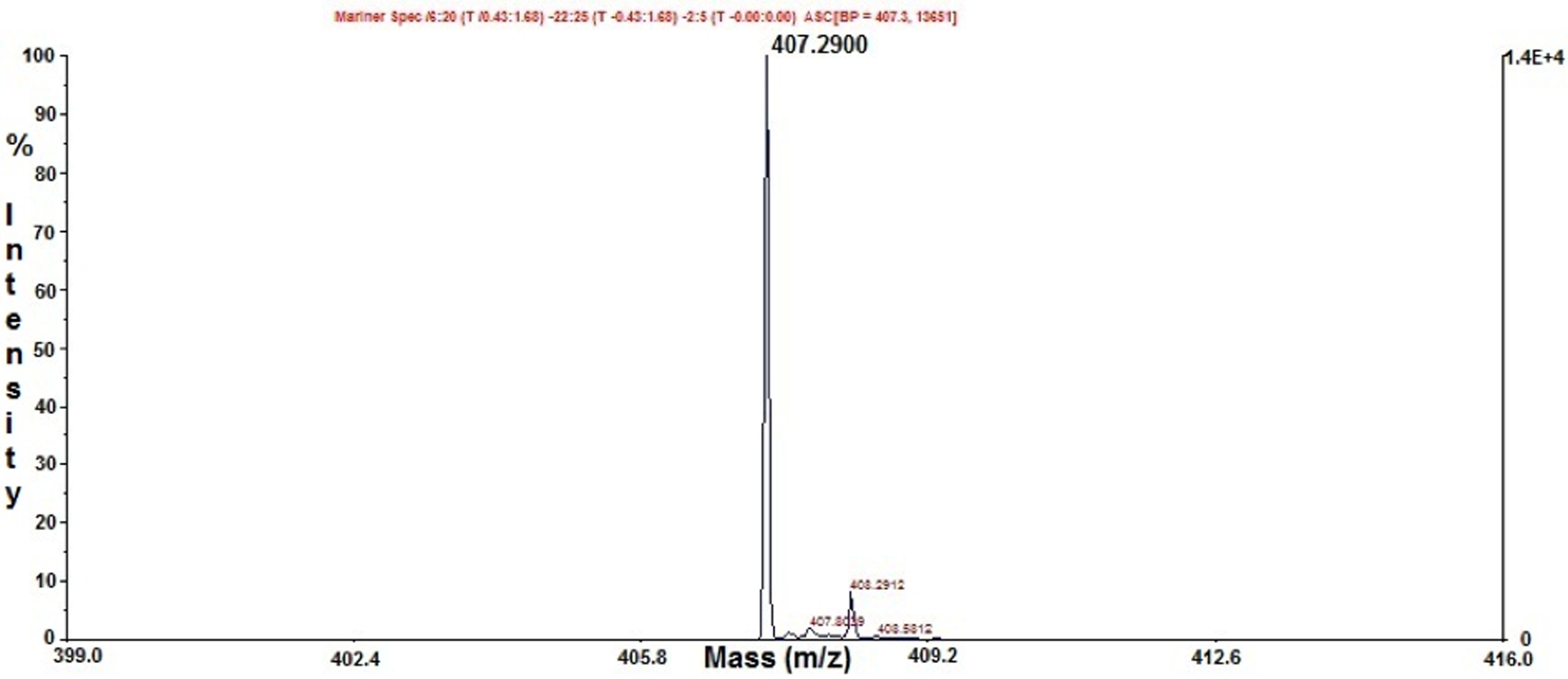


**Figure 1S.** Expanded region of ESI-TOF mass spectrum: component at t_R_=31.65 min in the chromatographic analysis of pulmonary leaflets decellularized with TriCOL. Experimental mass: 407.29 Da; Theoretical mass of COL: 407.60 Da (without Na^+^ ).


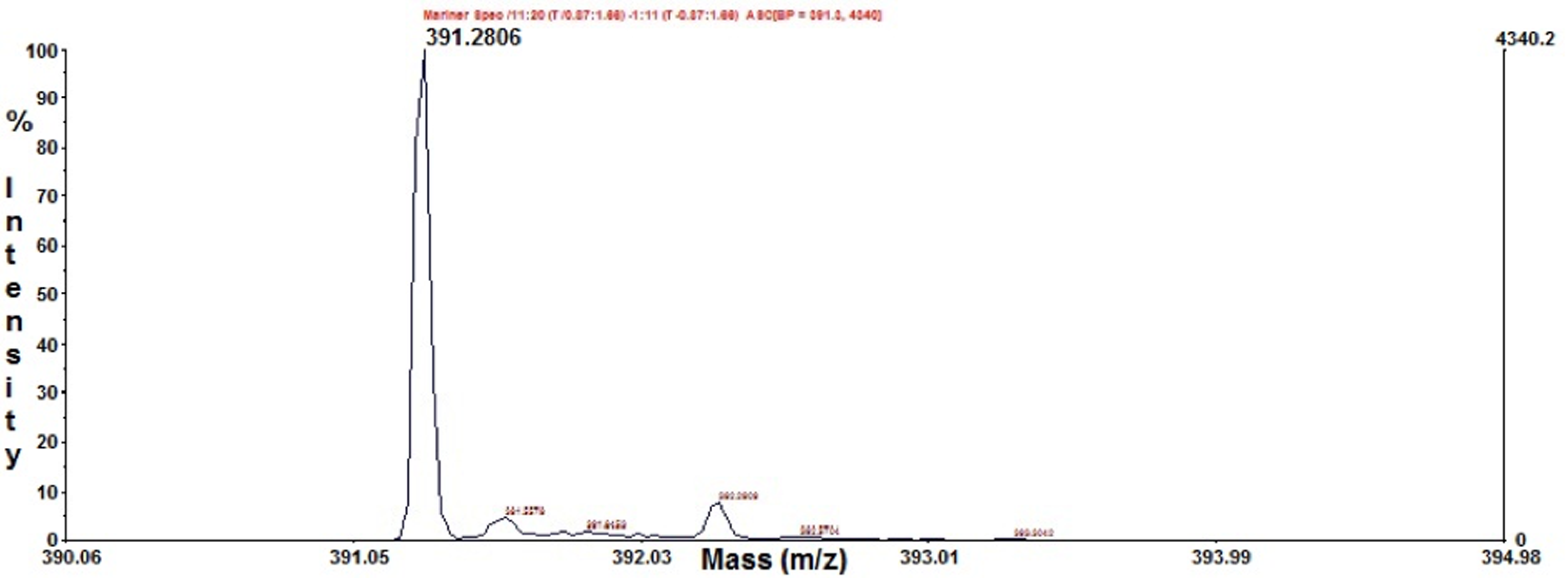


**Figure 2S.** Expanded region of ESI-TOF mass spectrum: component at t_R_=35.96 min in the chromatographic analysis of pulmonary leaflets decellularized with *S*-DOC. Experimental mass: 391.28 Da; Theoretical mass of DOC: 391.60 Da (without Na^+^ ).


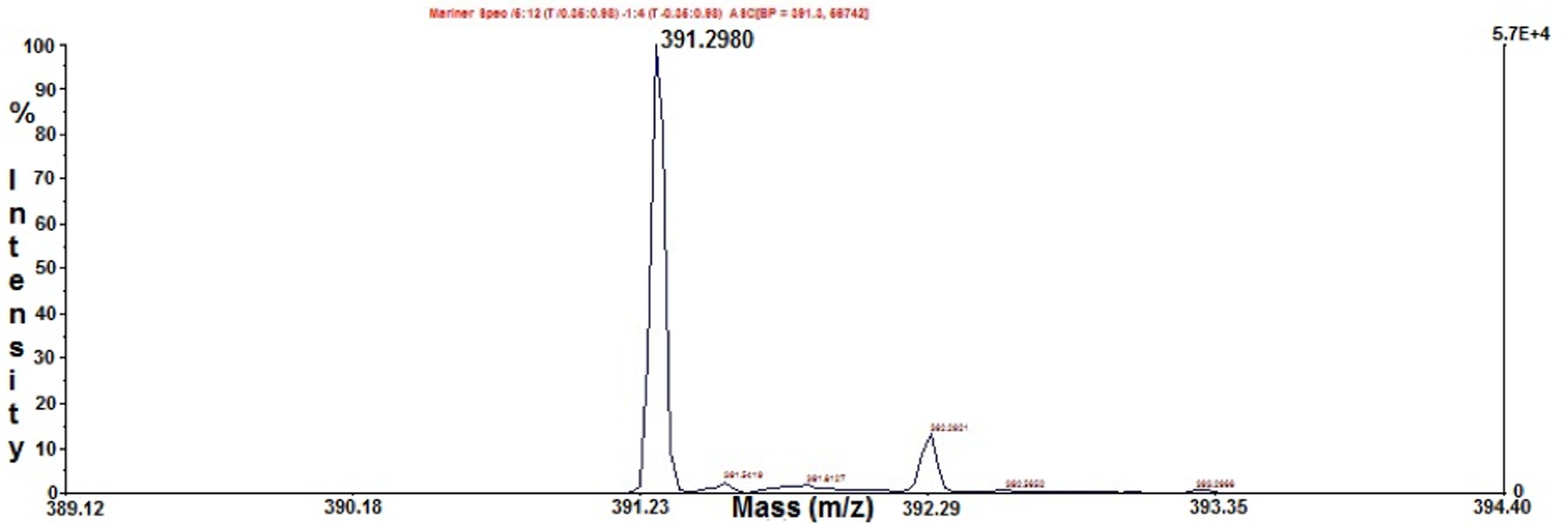


**Figure 3S.** Expanded region of ESI-TOF mass spectrum: component at t_R_=35.96 min in the chromatographic analysis of pulmonary leaflets decellularized with *C*-DOC/SDS. Experimental mass: 391.29 Da; Theoretical mass of DOC: 391.60 Da (without Na^+^).

**Figure 4S.** Expanded region of ESI-TOF mass spectrum: component at t_R_=27.50 min in the chromatographic analysis of pulmonary leaflets decellularized with TriTDOC. Experimental mass: 498.31 Da; Theoretical mass of TDOC: 498.69 Da (without Na^+^).
